# Study on relationship between pollen exine ornamentation pattern and germplasm evolution in flowering crabapple

**DOI:** 10.1038/srep39759

**Published:** 2017-01-06

**Authors:** Wang-Xiang Zhang, Ming-Ming Zhao, Jun-Jun Fan, Ting Zhou, Yong-Xia Chen, Fu-Liang Cao

**Affiliations:** 1 College of Forestry, Nanjing Forestry University, Nanjing 210037, China; 2 Co-Innovation Center for Sustainable Forestry in Southern China, Nanjing Forestry University, Nanjing 210037, China; 3 Yangzhou Crabapple Horticulture Company Limited, Yangzhou 225200, China; 4 School of Civil Engineering, Nanjing Forestry University, Nanjing 210037, China

## Abstract

Pollen ornamentation patterns are important in the study of plant genetic evolution and systematic taxonomy. However, they are normally difficult to quantify. Based on observations of pollen exine ornamentation characteristics of 128 flowering crabapple germplasms (44 natural species and 84 varieties), three qualitative variables with binary properties (X_i_: regularity of pollen exine ornamentation; Y_i_: scope of ornamentation arrangement regularity; Z_i_: ornamentation arrangement patterns) were extracted to establish a binary three-dimensional data matrix (X_i_ Y_i_ Z_i_) and the matrix data were converted to decimal data through weight assignment, which facilitated the unification of qualitative analysis and quantitative analysis. The result indicates that from species population to variety population and from parent population to variety population, the exine ornamentation of all three dimensions present the evolutionary trend of regular → irregular, wholly regular → partially regular, and single pattern → multiple patterns. Regarding the evolutionary degree, the regularity of ornamentation was significantly lower in both the variety population and progeny population, with a degree of decrease 0.82–1.27 times that of the regularity range of R-type ornamentation. In addition, the evolutionary degree significantly increased along X_i_  → Y_i_ → Z_i_. The result also has certain reference values for defining the taxonomic status of *Malus* species.

The flowering crabapples (*Malus* spp.) are a group of small landscape trees or shrubs. They have rich germplasm resources with charming flowers, colorful fruits and many tree shapes. Rehder’s study of the species and taxonomy of *Malus* Mill covered 25 species from all over the world and Li’s covered 26^1^,^2^. In Yu’s taxonomic report on Chinese species of *Malus* Mill, a total of 22 species were recognized^3^. However, the definitions of some species can be controversial because not all species have established characterization and classification criteria^4^. Hundreds of *Malus* Mill varieties have been developed over the course of its more than 200 year history of cultivation^5^,^6^, among which most varieties originated from selective breeding or occasional discovery and subsequently have unclear genetic backgrounds and inter-/intra-species crosses^7^. Although Jefferson identified ancestors for 181 *Malus* Mill varieties, the breeding pedigree for most of varieties is not yet clear^8^.

Pollen morphology has developed over the course of long-term evolution and shows species-specific characteristics^9^,^10^,^11^. Because pollen grain exine ornamentation is highly conserved and genetically stable^12^, it has been often used to investigate plant origin, genetic evolution and systematic taxonomy^12^,^13^,^14^. Based on the macro characteristics of pollen ornamentation, some studies have explored the evolutionary relationship between different ornamentations, such as reticulate, crisped, granular, or striated patterns^15^,^16^. However, these reports are limited to a qualitative descriptions, and evolution at this level is normally applicable to taxons above family and genus. With regard to taxons below genus, some works have explored the quantitative analysis method^17^,^18^,^19^ on genetics and evolution between germplasms based on local detailed features (ridge length and width, ridge interval, pore density and size) of pollen grain exine ornamentation. However, this method requires a great deal of measurement work and does not adequately reflect the overall feature of pollen grain exine ornamentation. According to the overall regularity of pollen ornamentation, Walker^20^, He and Hsu^21^ pointed out the evolutionary relationships between different ornamentation regularities, which still lacked quantitative support. So far, the studies of plant evolutionary relationships based on quantitative data of pollen ornamentation characteristics are seldom reported, because the pattern of pollen ornamentation is difficult to quantify directly. Pollen arrangement patterns are a comprehensive traits that cannot be reflected by a single index. Most indexes are qualitative traits rather than quantitative traits. The realization of the combination of the qualitative analysis and quantitative analysis is the key to analysis of pollen ornamentation characteristics.

This study extracted three variables on the basis of pollen ornamentation pattern characteristics to establish a binary three-dimensional data matrix (X_i_ Y_i_ Z_i_), and converted the matrix data to decimal data through weight assignment, in order to evaluate the degree of regularity of pollen ornamentation. This study aimed to (1) establish a simple and practical method of analyzing pollen grain evolutionary relationships, which can unify qualitative and quantitative analysis; (2) explore the evolutionary relationships (evolutionary direction and degree) between species population and variety population, and between parents population and progeny population. Results from our study will provide a new basis for taxonomy and status evaluation of flowering crabapple germplasm.

## Results

### Cluster analysis of pollen grain exine ornamentation regularity of flowering crabapple germplasm

Out of 131 flowering crabapple germplasms tested, only one species (*M. hupehensis*) and two varieties (*M*. ‘Strawberry Jelly’ and *M*. ‘Hydrangea’) had no pollen. All of the other 128 germplasms had pollen, including 44 species and 84 varieties.

**Figure 1:**
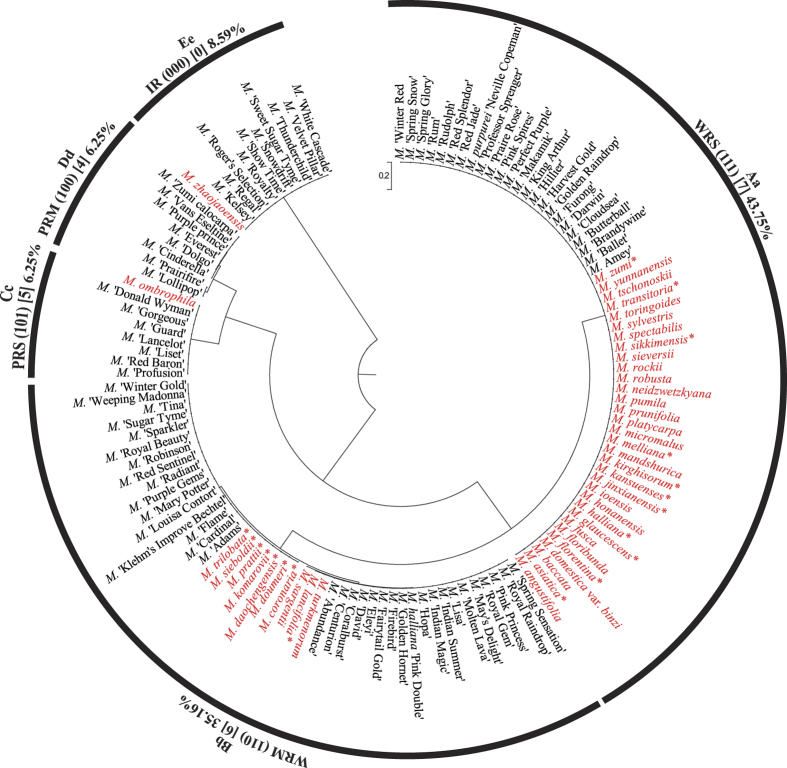
Cluster analysis of flower crabapple pollen grain exine ornamentation regularity. The red font represents stock species of flowering crabapple and the black font represents varieties. *Adapted from refs 21,29,31, 32, 33, 34, 35, 36, 37. WRS, Wholly Regular Single-pattern Group; WRM, Wholly Regular Multi-pattern Group; PRS, Partially Regular Single-pattern Group; PRM, Partially Regular Multi-pattern Group; IR, Irregular Group. Numerical values in square brackets, representing the score of each ornamentation group. Percentage, representing the germplasm quantity of each ornamentation group. Different lower case letters indicate significant differences at *p* < 0.05, and upper case letter indicate significant differences at *p* < 0.01 between scores of different ornamentation groups using Duncan’s test.

**Figure 2:**
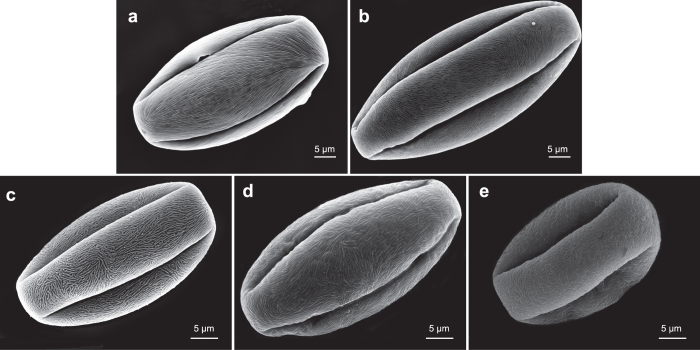
Representative scanning electron microscopic images of five types of flowering crabapple pollen exine ornamentation. (**a**) Wholly Regular Single-pattern Type (WRS), images of *Malus robusta* (×2500); (**b**) Wholly Regular Multi-pattern Type (WRM), images of *M. halliana* ‘Pink Double’ (×2500); (**c**) Partially Regular Single-pattern Type (PRS), images of *M*. ‘Red Baron’ (×3000); (**d**) Partially Regular Multi-pattern Type (PRM), images of *M*. ‘Everest’ (×3000); (**e**) Irregular Type (IR), images of *M*. ‘Velvet Pillar’ (×3000).

The three dimensional data matrix (X_i_ Y_i_ Z_i_) presented the degree of regularity of flowering crabapple pollen ornamentation. Cluster analysis of the tested flowering crabapple germplasm was conducted according to the three dimensional data of X_i_, Y_i_, Z_i_ (Fig. 1). When the Euclidean distance was 1.67, the 128 flowering crabapple germplasms were classified into five groups, WRS, WRM, PRS, PRM and IR. The entire pollen images of 108 tested germplasms can be found as Supplementary Fig. S1. Figure 2 provides the ornamentation arrangement characteristics. The germplasm quantitative distributions of the five groups are extremely unbalanced (with a variable coefficient of 0.90), and the germplasms are mainly distributed to WRS and WRM (79.0%). The degree of regularity of all groups of pollen ornamentation varied significantly (*p* = 0.0001) with scores of 7, 6, 5, 4, and 0, respectively.

### Regularity comparison of pollen grain exine ornamentation between species population and variety population of flowering crabapple

**Figure 3:**
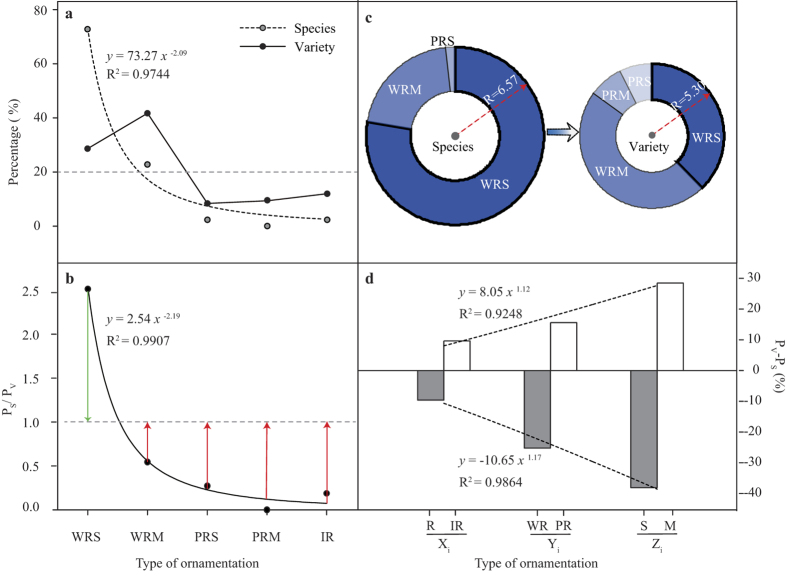
Comparison of pollen ornamentation regularities between species population and variety population of flowering crabapple. (**a**) The pollen ornamentation types’ constitution and weight distribution of species population and variety population of flowering crabapple. (**b**)The weighting ratio (P_S_/P_V_) distribution of ornamentation types in the two populations. Green column length (P_S_/P_V_ − 1) stands for the relative weight (the weight of WRS type in species population exceeds that in variety population); red column length (1 − P_S_/P_V_) represents the relative weight (the weight of pollen types in variety population exceeds that in species population). (**c**) The average score of pollen ornamentation degree of regularity of two populations and the weight of score of each ornamentation type. The radius of circle presents the average score value; the pie chart presents the weight constitution of the ornamentation types scores of pollen germplasms. (**d**) Weight differential of pollen ornamentations of the two populations in three dimensional directions (X_i_ Y_i_ Z_i_). P_S_, percentage of species quantity of each ornamentation type; P_V_, percentage of variety quantity of each ornamentation type. R, Regular Type; IR, Irregular Type; WR, Wholly Regular Type; PR, Partially Regular Type; S, Single-pattern Type; M, Multi-pattern Type.

Based on the cluster analysis in Fig. 1, the natural species population (44) and variety population (84) presented different pollen ornamentation types and weight distributions (Fig. 3a). The types showed different levels of regularity in their pollen ornamentation regularity, in descending order: WRS (score 7), WRM (score 6), PRS (score 5), PRM (score 4) and IR (score 0). Species population show a remarkable power function distribution with a unilaterally declining trend (R^2^ = 0.9744, *p* = 0.0017). Variety population show A-type distributions with a tendency to first increase and then decrease. In species and varieties, the distributions of the five types of pollen grain exine ornamentation were extremely unbalanced (with variable coefficients of 1.54 and 0.73, respectively). Variety had more balanced distributions than species population did (the variable coefficient was 0.47 times that of species population). For pollen ornamentation with a weight above 5%, species population had only WRS and WRM (the two types accounted for 95.5%), while varieties included all five types.

The weighting ratio (P_S_/P_V_) reflected the decrease in weight and decreasingly distant relationship between the pollen ornamentation types of the species and variety population. As shown in Fig. 3b, P_S_/P_V_ displays a significant power function distribution with a unilaterally declining trend (R^2^ = 0.9907, *p* = 0.0004). WRS: P_S_/P_V_ > 1 (P_S_/P_V_ = 1 + green column length), and the other four types: P_S_/P_V_ < 1 (P_S_/P_V_ = 1 − red column length, the red column gets longer from left to right), which indicated that the declining pollen weight of WRS of varieties is distributed to the other four types, and that less regularity in pollen ornamentation was associated with a greater increase in the range of weight. This trend of the weight was found to lead to a decrease in the regularity of pollen ornamentation in varieties. As shown in Fig. 3c, species population has higher score than varieties (6.57 vs. 5.30, *p* = 0.0004). The range of R-type pollen grain exine ornamentation was 1, which indicated that the overall decline in the regularity of ornamentation of varieties reached 1.27 times the range.

As shown in Fig. 3d, among the three dimensions of pollen ornamentation regularity matrix (X_i_ Y_i_ Z_i_), pollen weights of R-type, W-type, and S-type varieties were all lower than those of species population (ΔP = P_V_ − P_S_ < 0); while pollen weights of IR-type, P-type, and M-type varieties were all higher than those of species population (ΔP = P_V_ − P_S_ > 0). This indicates that the evolutionary trend of flowering crabapple pollen grain exine ornamentation was from regularity to irregularity (R → IR), from wholly regular to partially regular (WR → PR), and from single pattern to multiple patterns (S → M). The evolutionary degree showed a significant increasing trend as well (ΔP increases gradually) (R^2^ = 0.9864, *p* = 0.0321) along the three dimensional directions (X_i_ Y_i_ Z_i_).

### Comparison of pollen ornamentation regularities between parents and progeny of flowering crabapple

According to previous works^5^,^8^,^22^,^23^, 31 out of 84 tested flowering crabapple varieties can be partially or completely traced back to their parental germplasms (Table 1). The relationship between pollen ornamentation type and evolution has been analyzed at the individual and population levels.

Table 1 shows the relationship between pollen ornamentation type and evolution at the individual germplasm level. By converting trivariate matrix binary values to decimal values, each pollen ornamentation type was assigned a score that reflected the regularity of the ornamentation arrangement. Higher scores indicated more regular ornamentation. Results showed that progeny had regularity scores lower than that of their maximal-scoring parental germplasm.

According to pollen exine ornamentation regularity in descending order, the parental population had a remarkable power function distribution with a unilaterally declining trend (R^2^ = 0.9605, *p* = 0.0031). The progeny population showed an A-type distribution with a tendency to first increase and then decrease. Both in the parental and progeny population, the distributions of the five types of pollen ornamentation were extremely unbalanced (with the variable coefficient of 1.49 and 0.82, respectively). The progeny population showed a more balanced distribution than the parental population (the variable coefficient was 0.55 that of the parental population). The parental population was represented in only three types, WRS, WRM, and PRM, but the progeny population was represented in all five types.

**Figure 4:**
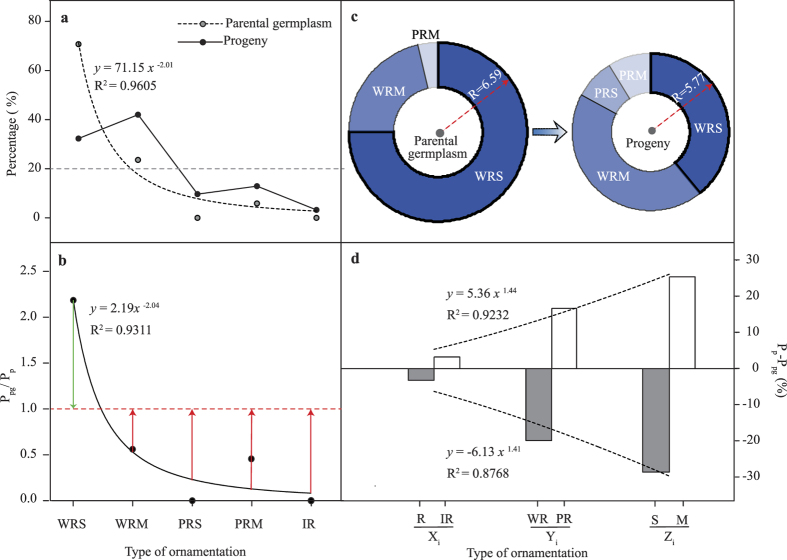
Comparison of pollen ornamentation regularities between parental population and progeny population. (**a**) The pollen ornamentation types’ constitution and weight distribution of parental population and progeny population of flowering crabapple. (**b**) The weighting ratio (P_Pg_/P_P_) distribution of ornamentation types in the two populations. Green column length (P_Pg_/P_P_ − 1) stands for the relative weight (the weight of WRS type in parental population exceeds that in progeny population); red column length (1 − P_Pg_/P_P_) stands for relative weight (the weight of pollen types in progeny population exceeds that in parental population). (**c**) The average score of pollen ornamentation degree of regularity of two populations and the weight of score of each type. The radius of circle presents the average score value; the pie chart presents the weight constitution of the ornamentation types scores of pollen germplasms. (**d**) Weight differential of pollen ornamentations of the two populations in three dimensional directions (X_i_ Y_i_ Z_i_). P_Pg_, percentage of parental germplasms quantity of each ornamentation type; P_P_, percentage of progeny quantity of each ornamentation type.

The weight growth and declining relationship between pollen ornamentation types of the two populations can be discerned by examining the weighting ratio (P_Pg_/P_P_) between the pollen grain exine ornamentation types of the parental and progeny populations. In Fig. 4b, P_Pg_/P_P_ shows a significant power function distribution with a unilaterally declining trend (*p* = 0.0066, R^2^ = 0.9311). WRS: P_Pg_/P_P_ > 1 P_Pg_/P_P_ = 1 + green column length), and the other four types: P_Pg_/P_P_ < 1 (P_Pg_/P_P_ = 1 − red column length, and the red column gets longer from left to right), which indicated that the lower pollen weight of the WRS type of the progeny population was distributed to the other four pollen types, and the lower pollen ornamentation regularity displayed a greater increase in weight. This weight changing trend leads to a reduction of pollen ornamentation regularity of the progeny population. As shown in Fig. 4c, the parental population showed higher scores than the progeny population (6.59 vs. 5.77, *p* = 0.0383), which reveals that the overall decline in regularity range of ornamentation of the progeny population reached 0.82 times that of the range of regularity in the ornamentation of R-type pollen.

As shown in Fig. 4d, in the three dimensions of the pollen ornamentation regularity matrix (X_i_ Y_i_ Z_i_), pollen weights of R-type, W-type, and S-type pollen of the progeny population were all lower than those of the parental population (ΔP = P_P_ − P_Pg_ < 0); while the pollen weights of IR-type, P-type, and M-type pollen of the progeny population were all higher than that of parental population (ΔP = P_P_ − P_Pg_ > 0). This indicated that the evolutionary trend of flowering crabapple pollen grain exine ornamentation was to move from regularity to irregularity (R → IR), from wholly regular to partially regular (WR → PR), and from single pattern to multiple patterns (S → M). The evolutionary degree also showed a significant increasing trend (ΔP increases gradually) (R^2^ = 0.9232; R^2^ = 0.8768) along the three dimensional directions of (X_i_ Y_i_ Z_i_).

## Discussion

Based on the overall pattern of pollen ornamentation arrangement, this study extracted a qualitative variable with binary properties (characteristics of yes or no) from three dimensions to establish a binary three-dimensional data matrix (X_i_ Y_i_ Z_i_), and converted the matrix data to decimal data through weight assignment, which indirectly established the quantification evaluation index for ascertaining the degree of regularity of pollen ornamentation. The index was easy to measure. It can not only present the evolutionary direction and degree of flowering crabapple pollen in three dimensions but alsoserves as a reference value for defining the taxonomic status of *Malus* species.

Pollen ornamentation arrangement patterns are important to the exploration of plant genetic evolution and systematic taxonomy. However, arrangement patterns are normally difficult to quantify. Walker investigated pollen ornamentation characteristics in 1000 species from 35 families and found that pollen grain exine ornamentation evolution to exhibit an overall exhibits a trend from regular to irregular and from simple to complicated^20^. He and Hsu investigated the pollen morphology of 26 species and 5 hybrids of the genus *Malus*^21^. They pointed out that the major evolutionary trend of striae arrangement was from regular and parallel to irregular, dense and interlocking. These researches were limited to a qualitative description of pollen ornamentation arrangement patterns, not quantification of these pattens, so there was no analysis of relationships among populations. Based on the overall pattern of pollen ornamentation arrangement, this study extracted three key qualitative variables (X_i_: regularity of pollen grain exine ornamentation; Y_i_: scope of ornamentation arrangement regularity; Z_i_: ornamentation arrangement modes) to establish a binary three-dimensional data matrix (X_i_ Y_i_ Z_i_), which revealed evolutionary patterns of flowering crabapple germplasm. In this way, the matrix variables combined overall information and local information for flowering crabapple pollen ornamentation, which had the advantages of high stability, strong degree of distinction, and easy measurement. Meanwhile, all of these informational variables adopted binary data of 0 and 1, and qualitative data could be converted to decimal quantitative data through weight assignment, to achieve the unification of qualitative and quantitative analyses. However, some information may be lost in the selection of key variables using this method. For example, the continuity of the striae is also an important indicator reflecting the overall characteristics of pollen ornamentation. Further improvement and optimization of this method are needed in further studies.

Second, the evolutionary direction and degree of flowering crabapple pollen in three dimensions can be revealed by utilizing the pollen ornamentation regularity matrix (X_i_ Y_i_ Z_i_). This study included an analysis of the evolutionary relationship of flowering crabapple pollen ornamentation at the individual and population levels. Through comparisons of the levels of regularity of pollen ornamentation between species population (44 pieces) and varieties (84 pieces), and between parental population (17 pieces) and progeny population (31 pieces) (population level), we found that regularity showed a marked decreasing trend (ranging from 0.82–1.27 times yhe range of regularity of ornamentation of R-type pollen). In the three dimensional directions of (X_i_ Y_i_ Z_i_), the evolutionary direction was R → IR, WR → PR, and S → M; the evolutionary degree increased significantly along X_i_  → Y_i_ → Z_i._ According to research on pollen ornamentation evolutionary trends between 17 parental populations and 31 corresponding progeny populations (individual level), results showed that no progeny had a degree of ornamentation greater than the maximal score of any of its parental germplasms, which suggested that there is a high degree of consistency between individual evolution and population evolution. These results are consistent with the results of research conducted by Walker^20^, He, and Hsu^21^. What makes this study different is that it not only describes the evolutionary direction of pollen ornamentation pattern, but also the evolutionary degree.

Third, pollen ornamentation patterns have certain reference values for defining the taxonomic status of *Malus* species. In this study, results showed that all of the *Malus* species had regular ornamentation (except for *M. zhaojaoensis*), although those with regular ornamentation were not necessarily species. In “Flowering Crab Apple”, some listed *Malus* species had controversial classification^4^, e.g., *M. floribunda, M. micromalus, M. platycarpa*, and *M. zumi*. Because the wild forms of *M. prunifolia* and *M. spectabilis* have not been discovered, Langenfelds^24^ and Wasson^25^ have questioned their germplasm status. Li^2^ suggested that *M. floribunda* may be a hybrid of *M. prunifolia* and *M. sieboldii*. Wasson^25^ considered *M. micromalus* to be a natural hybrid of *M. baccata* and *M. spectabilis, M. zumi* as the natural hybrid of *M. baccata* var. *mandshurica* and *M. sieboldii*, and *M. robusta* as the hybrid of *M. baccata* and *M. prunifolia*. McVaugh^26^ concluded that *M. platycarpa* is the hybrid of green apple and domesticated apple. Li *et al*.^27^ suggested that *M. toringoides* may be a natural hybrid of *M. transitoria* and *M. baccata*. However, in the Manual of Cultivated Trees and Shrubs^1^ and Chinese Fruit Taxonomy^28^, the species mentioned above were defined as *Malus* species that have been well-accepted by most researchers. Results showed that *M. platycarpa, M. sargentii, M. prunifolia, M. spectabilis, M. micromalus, M. floribunda*, and *M. zumi* all had the most regular ornamentation (WRS type) and that *M. toringoides* was also relatively regular but to a lesser extent (PRS type). The ornamentation of these controversial germplasm in the current study were consistent with the ornamentation characteristics of *Malus* species. In addition, *M. zhaojaoensis*, a new *Malus* species identified by Jiang^29^, was not included in the Plant List^30^. The present study showed it to have the most irregular ornamentation (IR type), which was not consistent with the ornamentation characteristics of species in general.

In conclusion, this study used three key variables to establish a binary three-dimensional data matrix (X_i_ Y_i_ Z_i_), and converted the matrix data to decimal data through weight assignment, which facilitated the unification of qualitative analysis and quantitative analysis and revealed the evolutionary direction and degree of flowering crabapple pollen in three dimensions. The evolutionary direction in three dimensions of (X_i_ Y_i_ Z_i_) was from regularity to irregularity (R → IR), from wholly regular to partially regular (WR → PR), and from single pattern to multiple patterns (S → M). Regarding the evolutionary degree, the regularity of ornamentation in flowering crabapple pollen displayed marked decreasing trends with a degree of decrease 0.82–1.27 times that of R-type pollen, and the evolution degree increased significantly along X_i_  → Y_i_ → Z_i._ The stock species of flowering crabapple have stronger regularity than other varieties, but strong regularity may not represent an individual species, which has certain reference value for defining taxonomic status of *Malus* species. The exact mechanism by which the regularity of pollen ornamentation arrangement showed a decreasing trend during the hybridization process requires further study. In addition, some information may be lost in the selection of key variables using data matrixes. If some dimensions were added (eg., the continuity of the striae), it would be advantageous to improve the accuracy of evolutionary analysis. Further improvement and optimization of this method are needed.

## Materials and Methods

### Materials

Detailed information regarding the testing of 131 flowering crabapple germplasms, including 45 stock species and 86 varieties, are listed in Table 2.

Pollen information regarding 20 flowering crabapple stock species was retrieved from the literature^21^,^31^,^32^,^33^,^34^,^35^,^36^,^37^,^38^. The other pollen samples from 111 flowering crabapple germplasms were collected from the Flowering Crabapple Germplasm Resources Garden, Nanjing Forestry University) (Jiangdu District, Yangzhou City, Jiangsu Province, China, Longitude 119°55′E, latitude 32°42′N). The age of flowering crabapple trees were between 5 and 8 years. During the early flowering stage, 20 flowers at the large bud stage were collected, wrapped in litmus paper, layered in a crisper at low temperature, and transferred to the lab on the same day. The anthers were peeled and air-dried before the following examination.

### FESEM observation of pollen and the characterization of pollen ornamentation

Type II Ultra-High Resolution Field Emission Scanning Electron Microscopy (Hitachi, S-4800) was used to observe pollen samples. The sample support was kept at room temperature and the acceleration voltage was 15 kV. Representative pollen grains were photographed.

**Figure 5:**
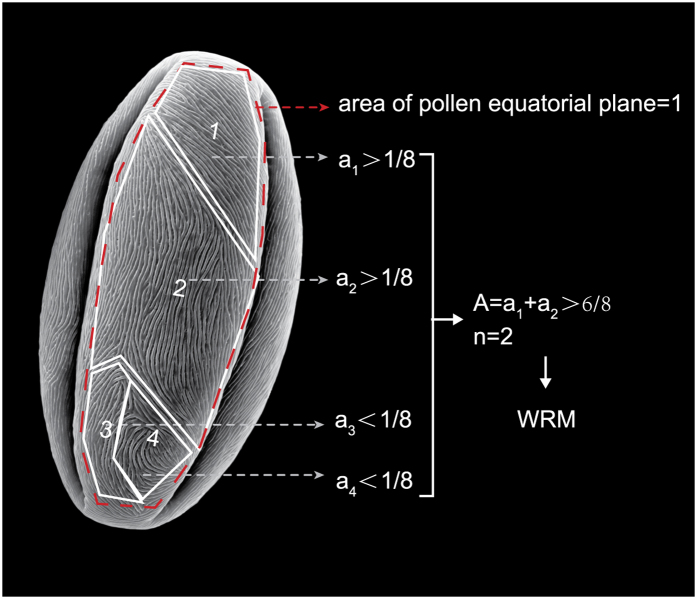
Illustration of judging the type of flowering crabapple pollen grain exine ornamentation (Wholly Regular Multi-pattern Type, WRM type as an example). 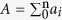
, (1/8 ≤ a_i_ ≤ 1, 0 ≤ n ≤ 8, 0 ≤ A ≤ 1). A represents the area of 1 unit with regularly arranged striae (relative area to the surface area of pollen equatorial plane, which was defined as 1; the size of the area by visual method.); n represents the number of units with regularly arranged striae where a ≥ 1/8; A represents the total area of all units with regularly arranged striae where a ≥ 1/8.

To evaluate the regularity of pollen grain ornamentation, a trivariate matrix (X_i_ Y_i_ Z_i_) was constructed to represent the ornamentation of the exine surface (the germination ditch and margins on the two polar ends were excluded). (1) Definition of X_i_: based on the degree of regularity of ornamentation, grains were classified as Regular Type (R) or Irregular Type (IR) and were assigned to binary values of X_i_ = 1 and 0, respectively. (2) Definition of Y_i_: based on the total area (A) of regularly arranged ornamentation, the grains were classified as Wholly Regular Type (WR) or Partially Regular Type (PR), and were assigned to binary values of Y_i_ = 1 and 0, respectively. (3) Definition of Z_i_: based on the number of ornamentation arrangement patterns, the grains were classified as Single-pattern Type (S) and Multi-pattern Type (M), and were assigned to binary values of Z_i_ = 1 and 0, respectively. As a result, all of the ornamentation of the exine surface of different flowering crabapple germplasms could be presented in a matrix form (X_i_ Y_i_ Z_i_) and could be divided into five major types: Wholly Regular Single-pattern Type (WRS, 1 1 1), Wholly Regular Multi-pattern Type (WRM, 1 1 0), Partially Regular Single-pattern Type (PRS, 1 0 1), Partially Regular Multi-pattern Type (PRM, 1 0 0), and Irregular Type (IR, 0 0 0). The illustration (Fig. 5) shows how the type of pollen grain exine ornamentation was judged. The binary values assignment criteria for the trivariate matrix (X_i_ Y_i_ Z_i_) are listed in Table 3.

### Data analysis

To analyze pollen grain ornamentation of the exine surface in different flowering crabapple germplasms, three variables were weighted based on importance. The order of the variables was regularity of ornamentation (X_i_) → total area of regularly arranged ornamentation (Y_i_) → pattern of ornamentation (Z_i_), or, X > Y > Z. The weight assignment method was based on a binary to decimal conversion. In brief, the number was written as 2^(n−1)^, in which n was the place number from right to left. Therefore, the binary to decimal conversion formula for the trivariate matrix was (X_i_ Y_i_ Z_i_) = X_i_ × 2^(3−1)^ + Y_i_ × 2^(2−1)^ + Z_i_ × 2^(1−1)^. Based on this formula, the scores for five major types were calculated as follows (Table 3):


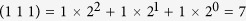



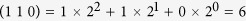



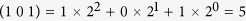



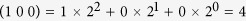



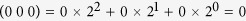


These scores reflected the regularity of grain ornamentation of the exine surface. A higher score represents a more regular ornamentation arrangement. When the scores are equal, lower n (the numbers of regularity ornamentation arrangement units) indicates stronger regularity.

ANOVA, Duncan’s test and Cluster analysis were conducted by SPSS 19.0. In order to improve the degree of distinction of matrix data during cluster analysis, Z_i_ = 1/n replacements were conducted at first, and then cluster analyses were conducted by directly adopting the 3D matrix data of (X_i_ Y_i_ Z_i_).

## Additional Information

**How to cite this article**: Zhang, W.-X. *et al*. Study on relationship between pollen exine ornamentation pattern and germplasm evolution in flowering crabapple. *Sci. Rep.* **7**, 39759; doi: 10.1038/srep39759 (2017).

**Publisher's note:** Springer Nature remains neutral with regard to jurisdictional claims in published maps and institutional affiliations.

## Supplementary Material

Supplementary Information

## Figures and Tables

**Table 1:**
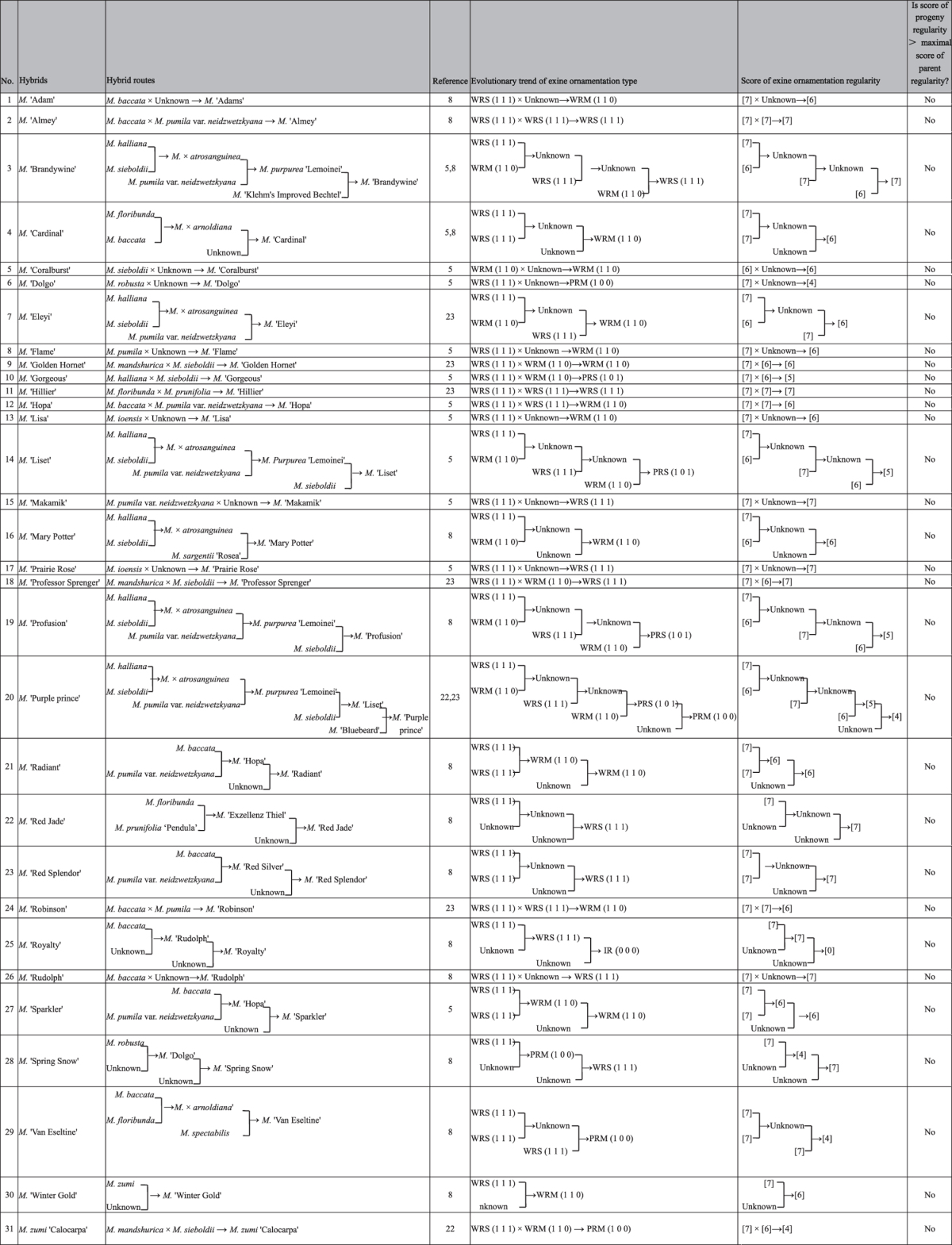
Parental traceability and exine sculpture type of pollen in flowering crabapple.

The ornamentation of pollen exine was presented in a matrix form (X_i_ Y_i_ Z_i_). WRS, (1 1 1); WRM, (1 1 0); PRS, (1 0 1); PRM, (1 0 0); IR, (0 0 0). The formula of score of exine ornamentation regularity: (X_i_ Y_i_Z_i_) = X_i_ × 2^(3−1)^ + Y_i_ × 2^(2−1)^ + Z_i_ × 2^(1−1)^.

**Table 2:** The list of flowering crabapple germplasms.

No. Species/varieties	No. Species/varieties	No. Species/varieties	No. Species/varieties
1 *Malus angustifolia*	34 *M. sieversii*	67 *M*. ‘Golden Hornet’	100 *M*. ‘Radiant’
2 *M. asiatica*^21^	35 *M. sikkimensis*^36^	68 *M*. ‘Golden Raindrop’	101 *M*. ‘Red Baron’
3 *M. baccata*	36 *M. spectabilis*	69 *M*. ‘Gorgeous’	102 *M*. ‘Red Jade’
4 *M. coronaria*^31^	37 *M. sylvestris*	70 *M*. ‘Guard’	103 *M*. ‘Red Sentinel’
5 *M. daochengensis*^21^	38 *M. toringoides*	71 *M. halliana* ‘Pink Double’	104 *M*. ‘Red Splendor’
6 *M. domestica* var. *binzi*	39 *M. transitoria*^31^	72 *M*. ‘Harvest Gold’	105 *M*. ‘Regal’
7 *M. doumeri*^33^	40 *M. trilobata*^31^	73 *M*. ‘Hillier’	106 *M*. ‘Robinson’
8 *M. florentina*^31^	41 *M. tschonoskii*	74 *M*. ‘Hopa’	107 *M*. ‘Roger’s Selection’
9 *M. floribunda*	42 *M. turkmenorum*	75 *M*. ‘Hydrangea’	108 *M*. ‘Royal Beauty’
10 *M. fusca*	43 *M. yunnanensis*	76 *M*. ‘Indian Magic’	109 *M*. ‘Royal Gem’
11 *M. glaucescens*^31^	44 *M. zhaojaoensis*^37^	77 *M*. ‘Indian Summer’	110 *M*. ‘Royal Raindrop’
12 *M. halliana*^32^	45 *M. zumi*^38^	78 *M*. ‘Kelsey’	111 *M*. ‘Royalty’
13 *M. honanensis*	46 *M*. ‘Abundance’	79 *M*. ‘King Arthur’	112 *M*. ‘Rudolph’
14 *M. hupehensis*	47 *M*. ‘Adams’	80 *M*. ‘Klehm’s Improve Bechtel’	113 *M*. ‘Rum’
15 *M. ioensis*	48 *M*. ‘Amey’	81 *M*. ‘Lancelot’	114 *M*. ‘Show Time’
16 *M. jinxianensis*^21^	49 *M*. ‘Ballet’	82 *M*. ‘Lisa’	115 *M*. ‘Snowdrift
17 *M. kansuensis*^31^	50 *M*. ‘Brandywine’	83 *M*. ‘Liset’	116 *M*. ‘Sparkler’
18 *M. kirghisorum*^33^	51 *M*. ‘Butterball’	84 *M*. ‘Lollipop’	117 *M*. ‘Spring Glory’
19 *M. komarovii*^21^	52 *M*. ‘Cardinal’	85 *M*. ‘Louisa Contort’	118 *M*. ‘Spring Sensation’
20 *M. lancifolia*^34^	53 *M*. ‘Centurion’	86 *M*. ‘Makamik’	119 *M*. ‘Spring Snow’
21 *M. mandshurica*	54 *M*. ‘Cinderella’	87 *M*. ‘Mary Potter’	120 *M*. ‘Strawberry Jelly’
22 *M. melliana*^35^	55 *M*. ‘Cloudsea’	88 *M*. ‘May’s Delight’	121 *M*. ‘Sugar Tyme’
23 *M. micromalus*	56 *M*. ‘Coralburst’	89 *M*. ‘Molten Lava’	122 *M*. ‘Sweet Sugar Tyme’
24 *M. ombrophila*	57 *M*. ‘Darwin’	90 *M*. ‘Perfect Purple’	123 *M*. ‘Thunderchild’
25 *M. platycarpa*	58 *M*. ‘David’	91 *M*. ‘Pink Princess’	124 *M*. ‘Tina’
26 *M. prattii*^21^	59 *M*. ‘Dolgo’	92 *M*. ‘Pink Spires’	125 *M*. ‘Vans Eseltine’
27 *M. prunifolia*	60 *M*. ‘Donald Wyman’	93 *M*. ‘Praire Rose’	126 *M*. ‘Velvet Pillar’
28 *M. pumila*	61 *M*. ‘Eleyi’	94 *M*. ‘Prairifire’	127 *M*. ‘Weeping Madonna’
29* M. pumila* var. *neidzwetzkyana*	62 *M*. ‘Everest’	95 *M*. ‘Professor Sprenger’	128 *M*. ‘White Cascade’
30 *M. robusta*	63 *M*. ‘Fairytail Gold’	96 *M*. ‘Profusion’	129 *M*. ‘Winter Gold’
31 *M. rockii*	64 *M*. ‘Firebird’	97 *M*. ‘Purple Gems’	130 *M*. ‘Winter Red
32 *M. sargentii*	65 *M*. ‘Flame’	98 *M*. ‘Purple prince’	131 *M. zumi* ‘Calocarpa’
33 *M. sieboldii*^31^	66 *M*. ‘Furong’	99 *M*. × *purpurei* ‘Neville Copeman’	

Superscript indicates the 20 flowering crabapple species whose pollen information were retrieved from the literature. The other 111 samples were collected from the Flowering Crabapple Germplasm Resources Garden, Nanjing Forestry University.

**Table 3:** Criterion for flowering crabapple pollen ornamentation type classification.

Type of ornamentation	Criterion	(X_i_ Y_i_ Z_i_)	Score
WRS	a ≥ 6/8	(1 1 1)	7
WRM	6/8 ≤ A, n > 1	(1 1 0)	6
PRS	3/8 ≤ A < 6/8, n = 1	(1 0 1)	5
PRM	3/8 ≤ A < 6/8, n > 1	(1 0 0)	4
IR	A < 3/8, or psilate smooth, unclear striae, reticulate or regulate ornamentation	(0 0 0)	0

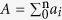
, (1/8 ≤ a_i_ ≤ 1, 0 ≤ n ≤ 8, 0 ≤ A ≤ 1). A represents the area of 1 unit with regularly arranged striae (relative area to the surface area of pollen equatorial plane, which was defined as 1; the size of the area by visual method.); n represents the number of units with regularly arranged striae where a ≥ 1/8; A represents the total area of all units with regularly arranged striae where a ≥ 1/8. The formula of score of exine sculpture type: (X_i_ Y_i_ Z_i_)  =  X_i_ × 2^(3−1)^ + Y_i_ × 2^(2−1)^ + Z_i_ × 2^(1−1)^.
