# Supplementary material for: Study on relationship between pollen exine ornamentation pattern and germplasm evolution in flowering crabapple
Source: Sci Rep. 2017 Jan 6;7:39759. doi: 10.1038/srep39759 (PMC5216378; doi:10.1038/srep39759)
Supplement: Supplementary Information [file srep39759-s1.pdf]

Title : Study on relationship between pollen exine ornamentation pattern and germplasm evolution in flowering crabapple

Wang-Xiang Zhang[1,2,3], Ming-Ming Zhao[1,2,3], Jun-Jun Fan[1,2], Ting Zhou[1,2], Yong-Xia Chen[1] & Fu-Liang Cao[1,2]

[1]College of Forestry, Nanjing Forestry University, Nanjing 210037, China. [2]Co-Innovation Center for Sustainable Forestry in Southern China, Nanjing Forestry University, Nanjing 210037, China. [3]Yangzhou Crabapple Horticulture Limited Company, Yangzhou 225200, China. Correspondence and requests for materials should be addressed to W.-X.Z. (email: malus2011@163.com)

Supplementary Figure S1 The pollen images of 108 flowering crabapple germplasms tested by SEM. **(a)** WRS, Wholly Regular Single-pattern Type; **(b)** WRM , Wholly Regular Multi-pattern Type; **(c)** PRS, Partially Regular Single-pattern Type; **(d)** PRM, Regular Multi-pattern Type; **(e)** IR , Irregular Type.

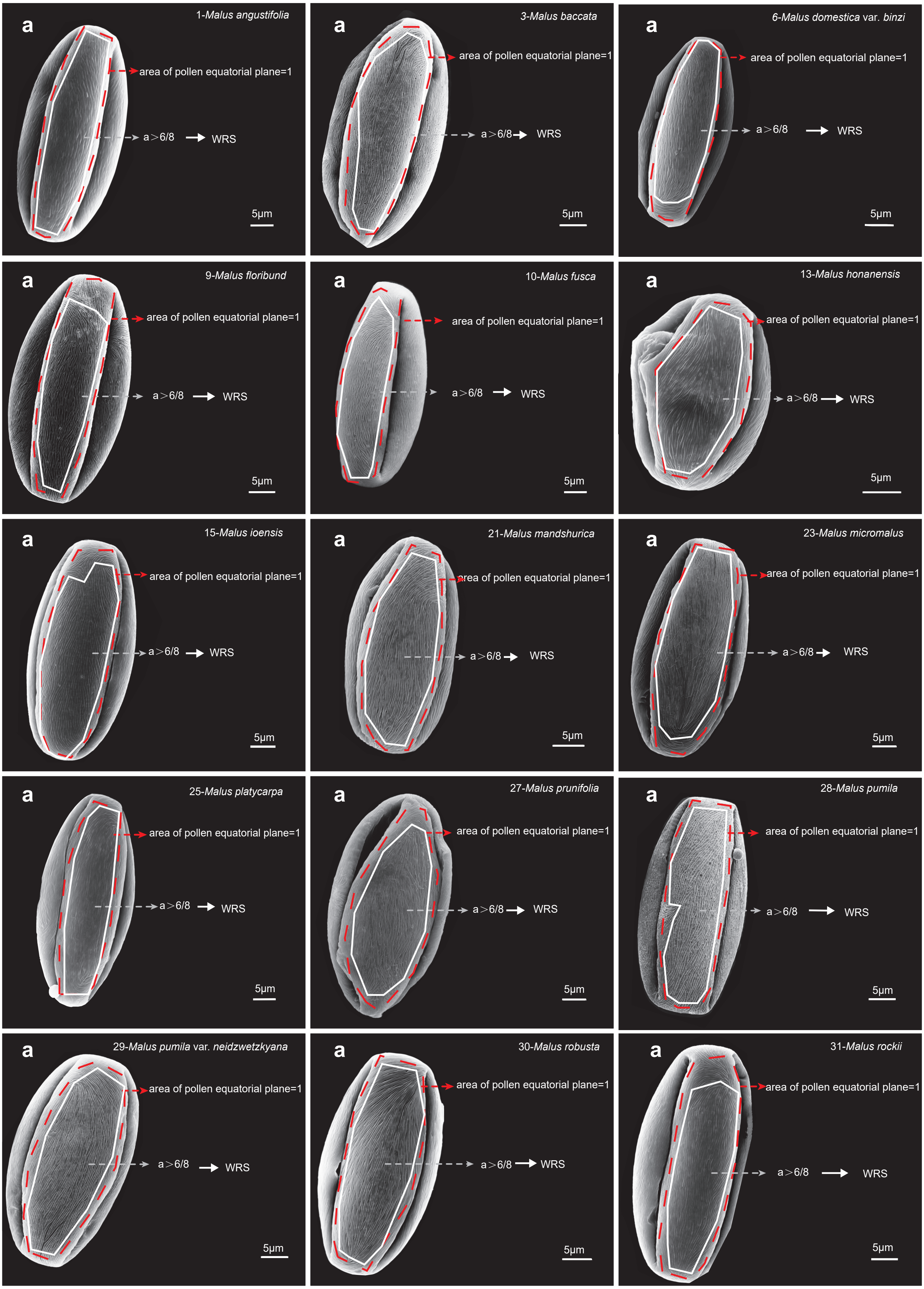

WRS (111) type:

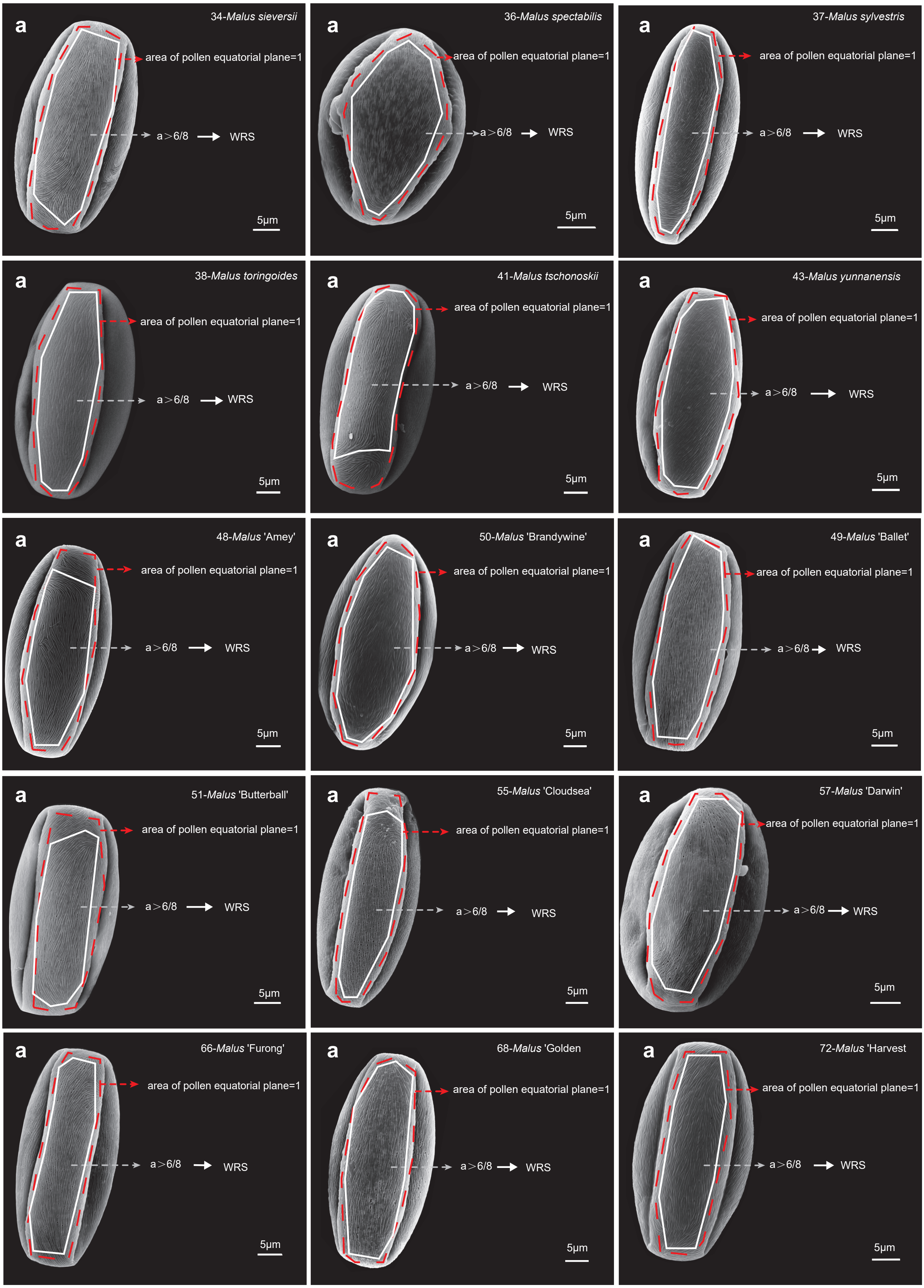
a
34-*Malus sieversii*
area of pollen equatorial plane=1
a>6/8
WRS
5μm
a
36-*Malus spectabilis*
area of pollen equatorial plane=1
a>6/8
WRS
5μm
a
37-*Malus sylvestris*
area of pollen equatorial plane=1
a>6/8
WRS
5μm
a
38-*Malus toringoides*
area of pollen equatorial plane=1
a>6/8
WRS
5μm
a
41-*Malus tschonoskii*
area of pollen equatorial plane=1
a>6/8
WRS
5μm
a
43-*Malus yunnanensis*
area of pollen equatorial plane=1
a>6/8
WRS
5μm
a
48-*Malus* 'Amey'
area of pollen equatorial plane=1
a>6/8
WRS
5μm
a
50-*Malus* 'Brandywine'
area of pollen equatorial plane=1
a>6/8
WRS
5μm
a
49-*Malus* 'Ballet'
area of pollen equatorial plane=1
a>6/8
WRS
5μm
a
51-*Malus* 'Butterball'
area of pollen equatorial plane=1
a>6/8
WRS
5μm
a
55-*Malus* 'Cloudsea'
area of pollen equatorial plane=1
a>6/8
WRS
5μm
a
57-*Malus* 'Darwin'
area of pollen equatorial plane=1
a>6/8
WRS
5μm
a
66-*Malus* 'Furong'
area of pollen equatorial plane=1
a>6/8
WRS
5μm
a
68-*Malus* 'Golden
area of pollen equatorial plane=1
a>6/8
WRS
5μm
a
72-*Malus* 'Harvest
area of pollen equatorial plane=1
a>6/8
WRS
5μm

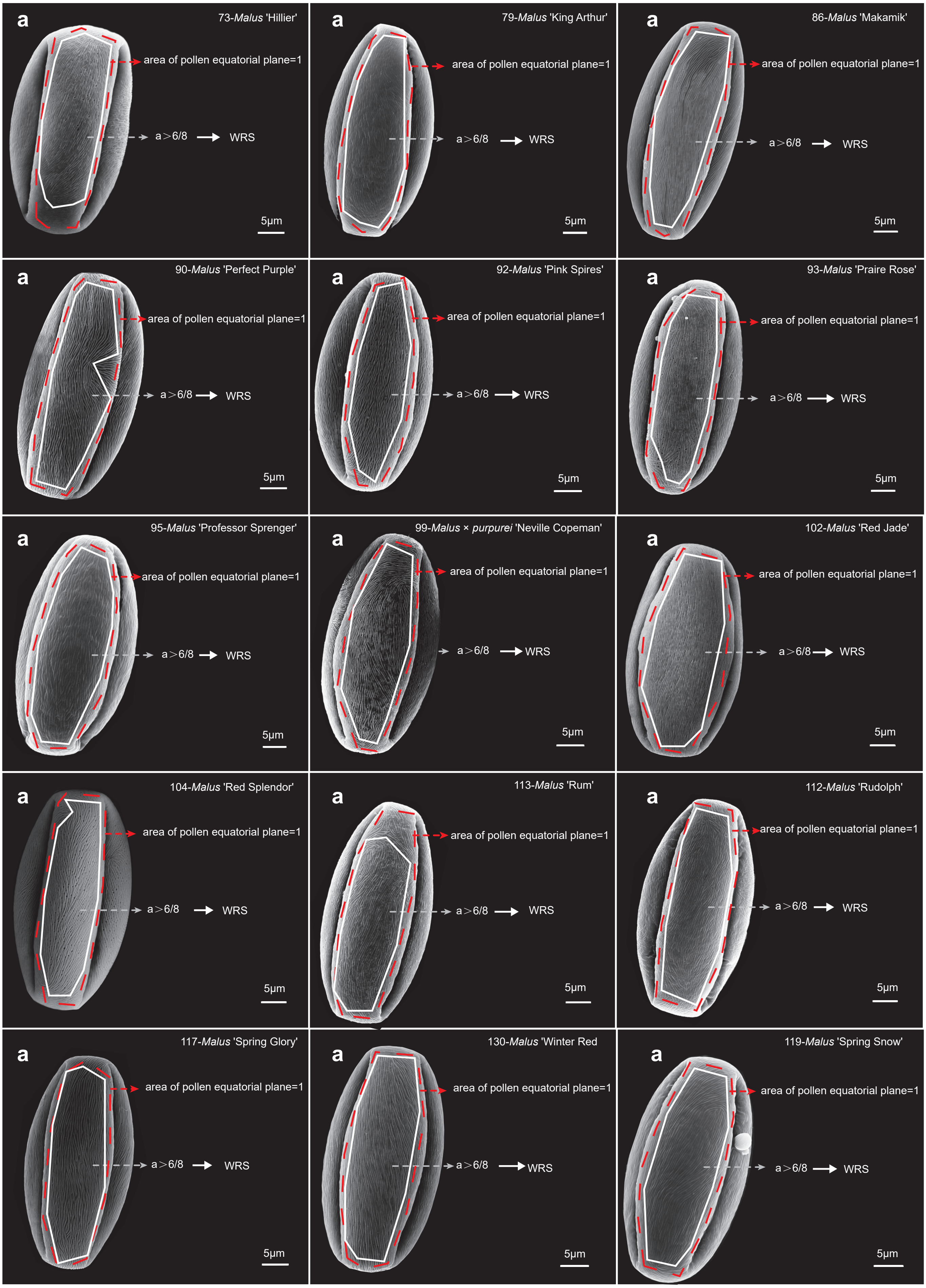
a
73-*Malus* 'Hillier'
area of pollen equatorial plane=1
a>6/8 → WRS
5μm
a
79-*Malus* 'King Arthur'
area of pollen equatorial plane=1
a>6/8 → WRS
5μm
a
86-*Malus* 'Makamik'
area of pollen equatorial plane=1
a>6/8 → WRS
5μm
a
90-*Malus* 'Perfect Purple'
area of pollen equatorial plane=1
a>6/8 → WRS
5μm
a
92-*Malus* 'Pink Spires'
area of pollen equatorial plane=1
a>6/8 → WRS
5μm
a
93-*Malus* 'Praire Rose'
area of pollen equatorial plane=1
a>6/8 → WRS
5μm
a
95-*Malus* 'Professor Sprenger'
area of pollen equatorial plane=1
a>6/8 → WRS
5μm
a
99-*Malus* × *purpurei* 'Neville Copeman'
area of pollen equatorial plane=1
a>6/8 → WRS
5μm
a
102-*Malus* 'Red Jade'
area of pollen equatorial plane=1
a>6/8 → WRS
5μm
a
104-*Malus* 'Red Splendor'
area of pollen equatorial plane=1
a>6/8 → WRS
5μm
a
113-*Malus* 'Rum'
area of pollen equatorial plane=1
a>6/8 → WRS
5μm
a
112-*Malus* 'Rudolph'
area of pollen equatorial plane=1
a>6/8 → WRS
5μm
a
117-*Malus* 'Spring Glory'
area of pollen equatorial plane=1
a>6/8 → WRS
5μm
a
130-*Malus* 'Winter Red
area of pollen equatorial plane=1
a>6/8 → WRS
5μm
a
119-*Malus* 'Spring Snow'
area of pollen equatorial plane=1
a>6/8 → WRS
5μm

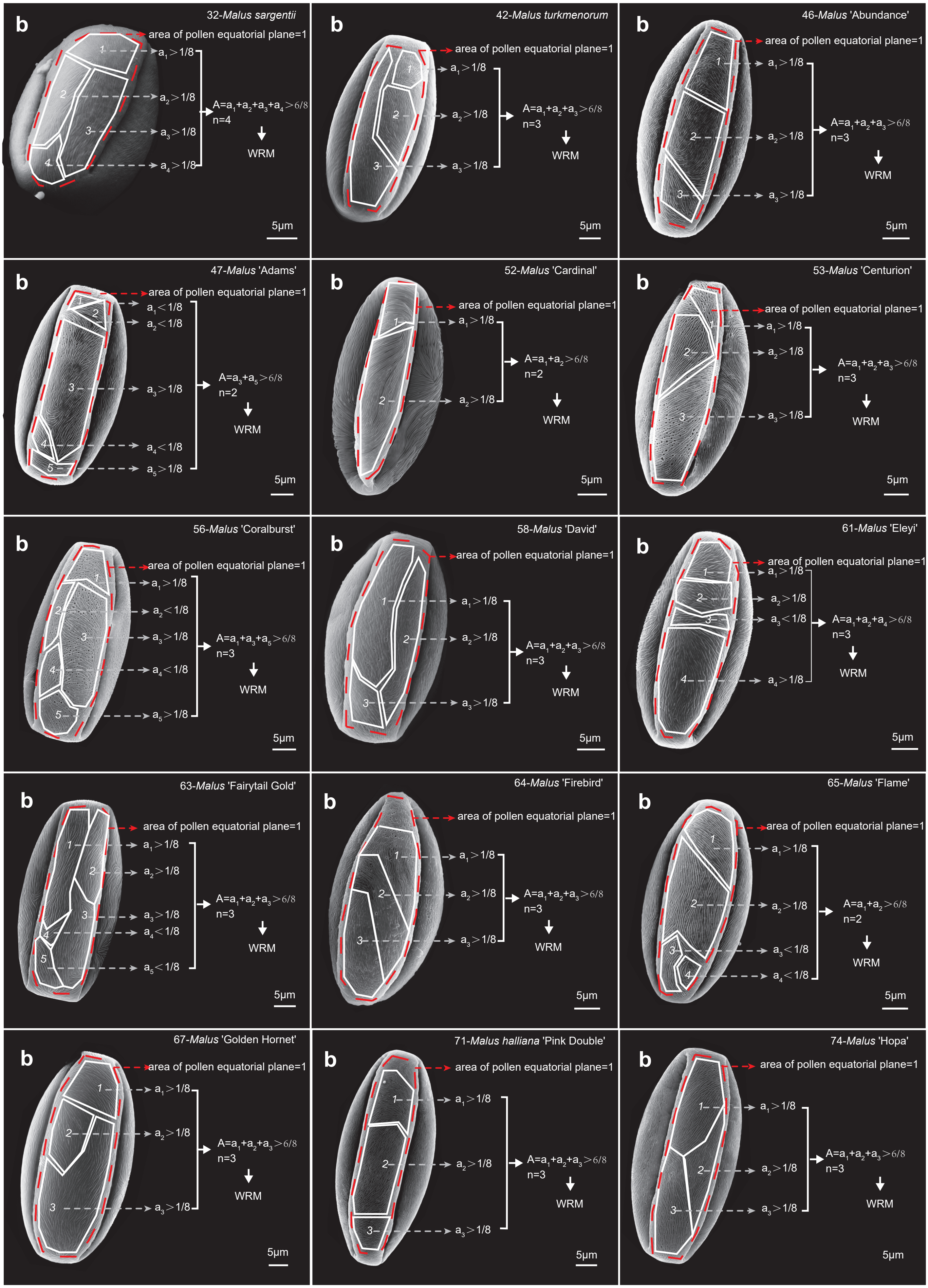
b
32-Malus sargentii
area of pollen equatorial plane=1
a1>1/8
a2>1/8
a3>1/8
a4>1/8
A=a1+a2+a3+a4>6/8
n=4
WRM
5μm
b
42-Malus turkmenorum
area of pollen equatorial plane=1
a1>1/8
a2>1/8
a3>1/8
A=a1+a2+a3>6/8
n=3
WRM
5μm
b
46-Malus 'Abundance'
area of pollen equatorial plane=1
a1>1/8
a2>1/8
a3>1/8
A=a1+a2+a3>6/8
n=3
WRM
5μm
b
47-Malus 'Adams'
area of pollen equatorial plane=1
a1<1/8
a2<1/8
a3>1/8
a4<1/8
a5>1/8
A=a3+a5>6/8
n=2
WRM
5μm
b
52-Malus 'Cardinal'
area of pollen equatorial plane=1
a1>1/8
a2>1/8
A=a1+a2>6/8
n=2
WRM
5μm
b
53-Malus 'Centurion'
area of pollen equatorial plane=1
a1>1/8
a2>1/8
a3>1/8
A=a1+a2+a3>6/8
n=3
WRM
5μm
b
56-Malus 'Coralburst'
area of pollen equatorial plane=1
a1>1/8
a2<1/8
a3>1/8
a4<1/8
a5>1/8
A=a1+a3+a5>6/8
n=3
WRM
5μm
b
58-Malus 'David'
area of pollen equatorial plane=1
a1>1/8
a2>1/8
a3>1/8
A=a1+a2+a3>6/8
n=3
WRM
5μm
b
61-Malus 'Eleyi'
area of pollen equatorial plane=1
a1>1/8
a2>1/8
a3<1/8
a4>1/8
A=a1+a2+a4>6/8
n=3
WRM
5μm
b
63-Malus 'Fairytail Gold'
area of pollen equatorial plane=1
a1>1/8
a2>1/8
a3>1/8
a4<1/8
a5<1/8
A=a1+a2+a3>6/8
n=3
WRM
5μm
b
64-Malus 'Firebird'
area of pollen equatorial plane=1
a1>1/8
a2>1/8
a3>1/8
A=a1+a2+a3>6/8
n=3
WRM
5μm
b
65-Malus 'Flame'
area of pollen equatorial plane=1
a1>1/8
a2>1/8
a3<1/8
a4<1/8
A=a1+a2>6/8
n=2
WRM
5μm
b
67-Malus 'Golden Hornet'
area of pollen equatorial plane=1
a1>1/8
a2>1/8
a3>1/8
A=a1+a2+a3>6/8
n=3
WRM
5μm
b
71-Malus halliana 'Pink Double'
area of pollen equatorial plane=1
a1>1/8
a2>1/8
a3>1/8
A=a1+a2+a3>6/8
n=3
WRM
5μm
b
74-Malus 'Hopa'
area of pollen equatorial plane=1
a1>1/8
a2>1/8
a3>1/8
A=a1+a2+a3>6/8
n=3
WRM
5μm

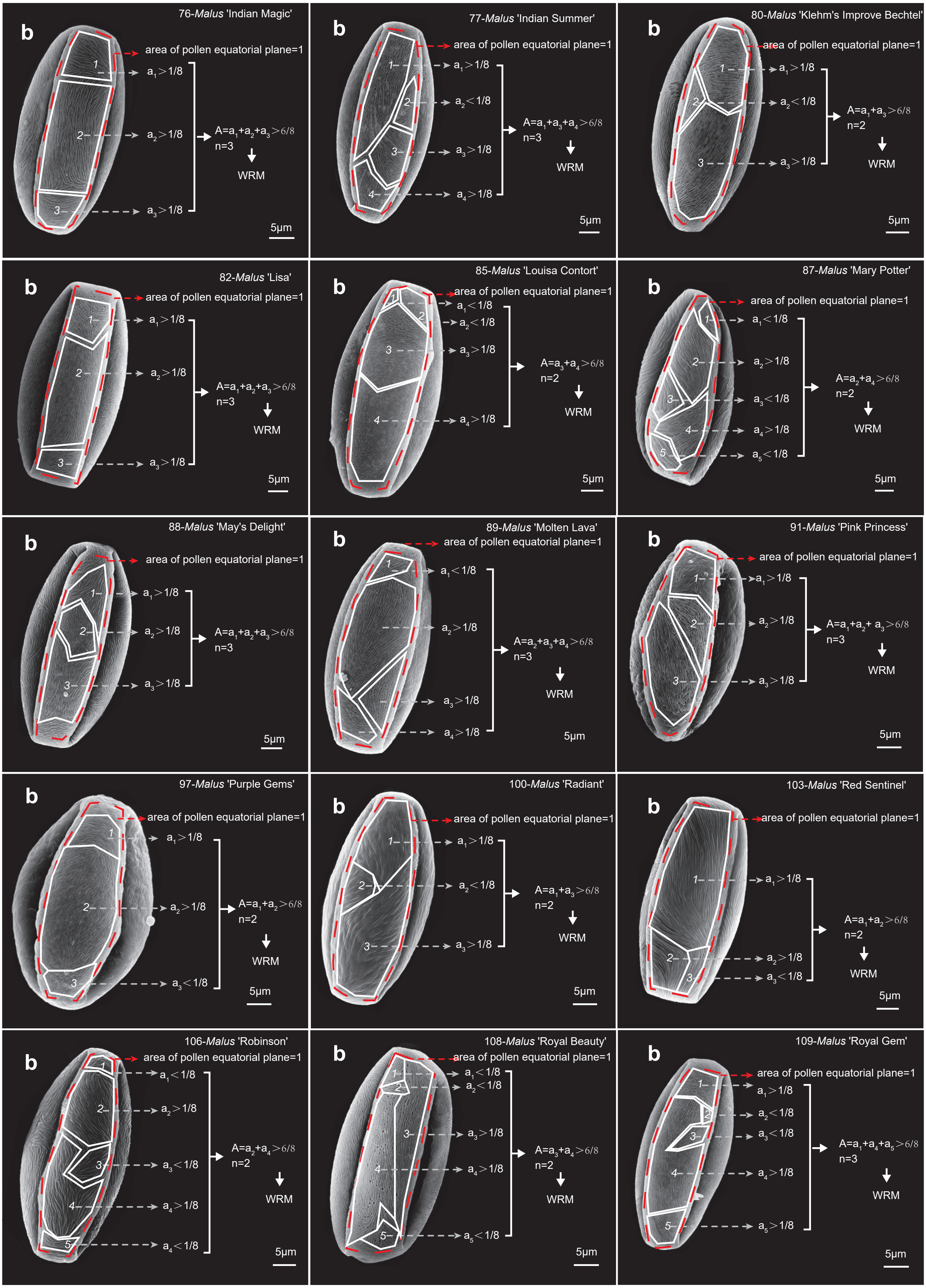
b
76-*Malus* 'Indian Magic'
area of pollen equatorial plane=1
$a_1>1/8$
$a_2>1/8$
$a_3>1/8$
$A=a_1+a_2+a_3>6/8$
n=3
WRM
5μm
b
77-*Malus* 'Indian Summer'
area of pollen equatorial plane=1
$a_1>1/8$
$a_2<1/8$
$a_3>1/8$
$a_4>1/8$
$A=a_1+a_3+a_4>6/8$
n=3
WRM
5μm
b
80-*Malus* 'Klehm's Improve Bechtel'
area of pollen equatorial plane=1
$a_1>1/8$
$a_2<1/8$
$a_3>1/8$
$A=a_1+a_3>6/8$
n=2
WRM
5μm
b
82-*Malus* 'Lisa'
area of pollen equatorial plane=1
$a_1>1/8$
$a_2>1/8$
$a_3>1/8$
$A=a_1+a_2+a_3>6/8$
n=3
WRM
5μm
b
85-*Malus* 'Louisa Contort'
area of pollen equatorial plane=1
$a_1<1/8$
$a_2<1/8$
$a_3>1/8$
$a_4>1/8$
$A=a_3+a_4>6/8$
n=2
WRM
5μm
b
87-*Malus* 'Mary Potter'
area of pollen equatorial plane=1
$a_1<1/8$
$a_2>1/8$
$a_3<1/8$
$a_4>1/8$
$a_5<1/8$
$A=a_2+a_4>6/8$
n=2
WRM
5μm
b
88-*Malus* 'May's Delight'
area of pollen equatorial plane=1
$a_1>1/8$
$a_2>1/8$
$a_3>1/8$
$A=a_1+a_2+a_3>6/8$
n=3
5μm
b
89-*Malus* 'Molten Lava'
area of pollen equatorial plane=1
$a_1<1/8$
$a_2>1/8$
$a_3>1/8$
$a_4>1/8$
$A=a_2+a_3+a_4>6/8$
n=3
WRM
5μm
b
91-*Malus* 'Pink Princess'
area of pollen equatorial plane=1
$a_1>1/8$
$a_2>1/8$
$a_3>1/8$
$A=a_1+a_2+a_3>6/8$
n=3
WRM
5μm
b
97-*Malus* 'Purple Gems'
area of pollen equatorial plane=1
$a_1>1/8$
$a_2>1/8$
$a_3<1/8$
$A=a_1+a_2>6/8$
n=2
WRM
5μm
b
100-*Malus* 'Radiant'
area of pollen equatorial plane=1
$a_1>1/8$
$a_2<1/8$
$a_3>1/8$
$A=a_1+a_3>6/8$
n=2
WRM
5μm
b
103-*Malus* 'Red Sentinel'
area of pollen equatorial plane=1
$a_1>1/8$
$a_2>1/8$
$a_3<1/8$
$A=a_1+a_2>6/8$
n=2
WRM
5μm
b
106-*Malus* 'Robinson'
area of pollen equatorial plane=1
$a_1<1/8$
$a_2>1/8$
$a_3<1/8$
$a_4>1/8$
$a_4<1/8$
$A=a_2+a_4>6/8$
n=2
WRM
5μm
b
108-*Malus* 'Royal Beauty'
area of pollen equatorial plane=1
$a_1<1/8$
$a_2<1/8$
$a_3>1/8$
$a_4>1/8$
$a_5<1/8$
$A=a_3+a_4>6/8$
n=2
WRM
5μm
b
109-*Malus* 'Royal Gem'
area of pollen equatorial plane=1
$a_1>1/8$
$a_2<1/8$
$a_3<1/8$
$a_4>1/8$
$a_5>1/8$
$A=a_1+a_4+a_5>6/8$
n=3
WRM
5μm

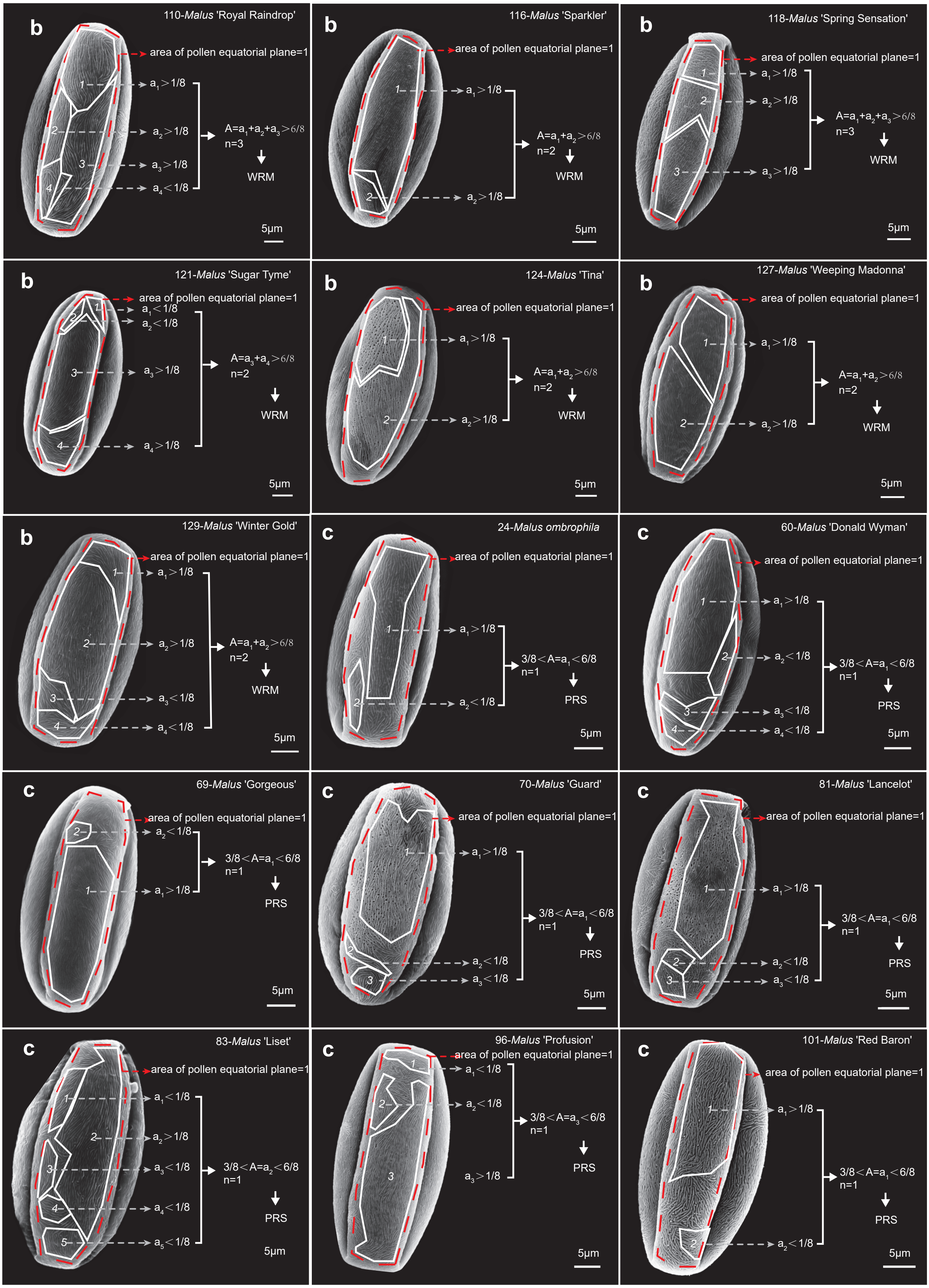

b
110-*Malus* 'Royal Raindrop'
area of pollen equatorial plane=1
$a_1>1/8$
$a_2>1/8$
$a_3>1/8$
$a_4<1/8$
$A=a_1+a_2+a_3>6/8$
n=3
WRM
5μm
b
116-*Malus* 'Sparkler'
area of pollen equatorial plane=1
$a_1>1/8$
$a_2>1/8$
$A=a_1+a_2>6/8$
n=2
WRM
5μm
b
118-*Malus* 'Spring Sensation'
area of pollen equatorial plane=1
$a_1>1/8$
$a_2>1/8$
$a_3>1/8$
$A=a_1+a_2+a_3>6/8$
n=3
WRM
5μm
b
121-*Malus* 'Sugar Tyme'
area of pollen equatorial plane=1
$a_1<1/8$
$a_2<1/8$
$a_3>1/8$
$a_4>1/8$
$A=a_3+a_4>6/8$
n=2
WRM
5μm
b
124-*Malus* 'Tina'
area of pollen equatorial plane=1
$a_1>1/8$
$a_2>1/8$
$A=a_1+a_2>6/8$
n=2
WRM
5μm
b
127-*Malus* 'Weeping Madonna'
area of pollen equatorial plane=1
$a_1>1/8$
$a_2>1/8$
$A=a_1+a_2>6/8$
n=2
WRM
5μm
b
129-*Malus* 'Winter Gold'
area of pollen equatorial plane=1
$a_1>1/8$
$a_2>1/8$
$a_3<1/8$
$a_4<1/8$
$A=a_1+a_2>6/8$
n=2
WRM
5μm
c
24-*Malus ombrophila*
area of pollen equatorial plane=1
$a_1>1/8$
$a_2<1/8$
$3/8<A=a_1<6/8$
n=1
PRS
5μm
c
60-*Malus* 'Donald Wyman'
area of pollen equatorial plane=1
$a_1>1/8$
$a_2<1/8$
$a_3<1/8$
$a_4<1/8$
$3/8<A=a_1<6/8$
n=1
PRS
5μm
c
69-*Malus* 'Gorgeous'
area of pollen equatorial plane=1
$a_2<1/8$
$a_1>1/8$
$3/8<A=a_1<6/8$
n=1
PRS
5μm
c
70-*Malus* 'Guard'
area of pollen equatorial plane=1
$a_1>1/8$
$a_2<1/8$
$a_3<1/8$
$3/8<A=a_1<6/8$
n=1
PRS
5μm
c
81-*Malus* 'Lancelot'
area of pollen equatorial plane=1
$a_1>1/8$
$a_2<1/8$
$a_3<1/8$
$3/8<A=a_1<6/8$
n=1
PRS
5μm
c
83-*Malus* 'Liset'
area of pollen equatorial plane=1
$a_1<1/8$
$a_2>1/8$
$a_3<1/8$
$a_4<1/8$
$a_5<1/8$
$3/8<A=a_2<6/8$
n=1
PRS
5μm
c
96-*Malus* 'Profusion'
area of pollen equatorial plane=1
$a_1<1/8$
$a_2<1/8$
$a_3>1/8$
$3/8<A=a_3<6/8$
n=1
PRS
5μm
c
101-*Malus* 'Red Baron'
area of pollen equatorial plane=1
$a_1>1/8$
$a_2<1/8$
$3/8<A=a_1<6/8$
n=1
PRS
5μm

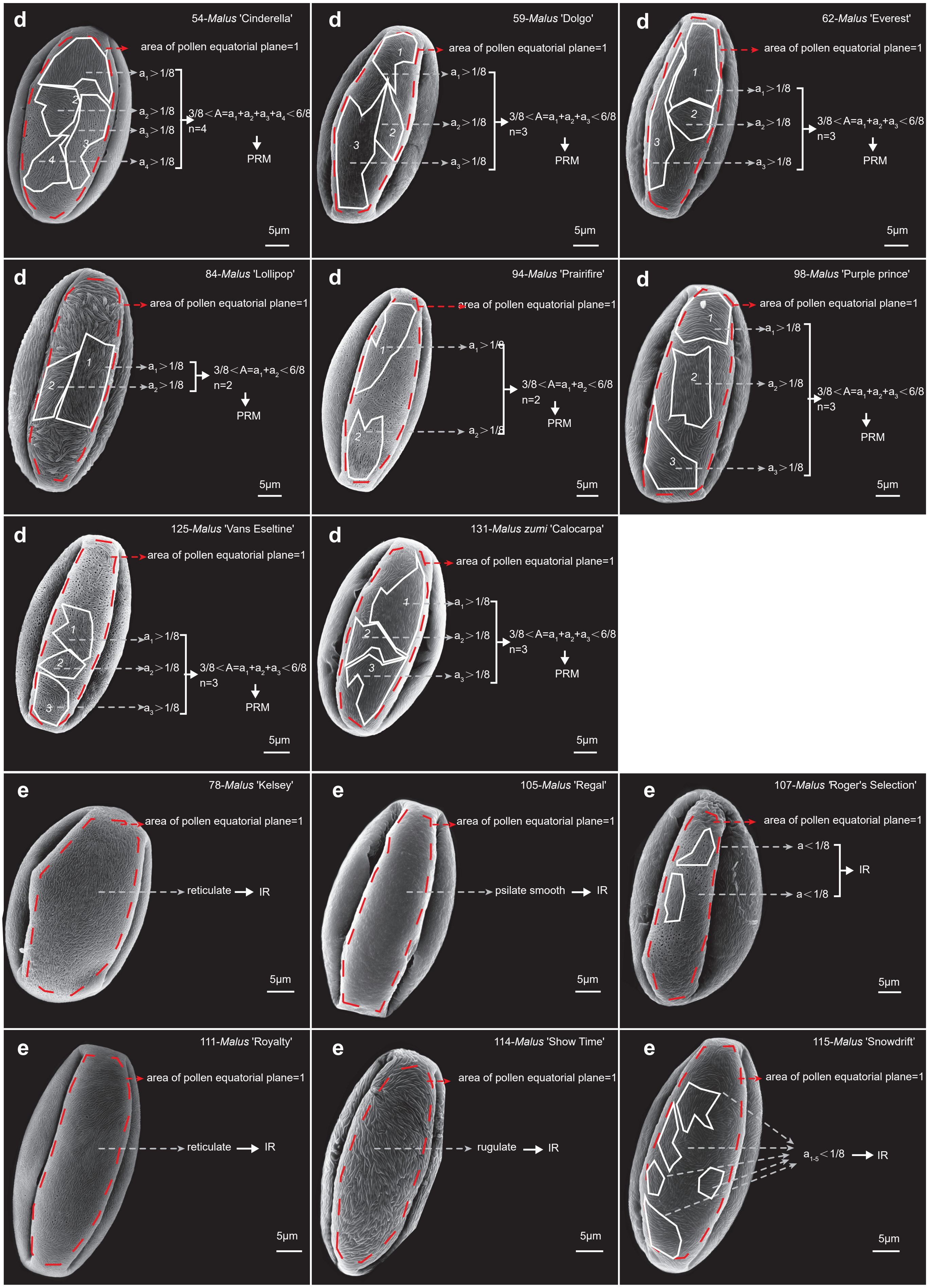
d
54-*Malus* 'Cinderella'
area of pollen equatorial plane=1
$a_1>1/8$
$a_2>1/8$
$a_3>1/8$
$a_4>1/8$
$3/8<A=a_1+a_2+a_3+a_4<6/8$
n=4
PRM
5μm
d
59-*Malus* 'Dolgo'
area of pollen equatorial plane=1
$a_1>1/8$
$a_2>1/8$
$a_3>1/8$
$3/8<A=a_1+a_2+a_3<6/8$
n=3
PRM
5μm
d
62-*Malus* 'Everest'
area of pollen equatorial plane=1
$a_1>1/8$
$a_2>1/8$
$a_3>1/8$
$3/8<A=a_1+a_2+a_3<6/8$
n=3
PRM
5μm
d
84-*Malus* 'Lollipop'
area of pollen equatorial plane=1
$a_1>1/8$
$a_2>1/8$
$3/8<A=a_1+a_2<6/8$
n=2
PRM
5μm
d
94-*Malus* 'Prairifire'
area of pollen equatorial plane=1
$a_1>1/8$
$a_2>1/8$
$3/8<A=a_1+a_2<6/8$
n=2
PRM
5μm
d
98-*Malus* 'Purple prince'
area of pollen equatorial plane=1
$a_1>1/8$
$a_2>1/8$
$a_3>1/8$
$3/8<A=a_1+a_2+a_3<6/8$
n=3
PRM
5μm
d
125-*Malus* 'Vans Eseltine'
area of pollen equatorial plane=1
$a_1>1/8$
$a_2>1/8$
$a_3>1/8$
$3/8<A=a_1+a_2+a_3<6/8$
n=3
PRM
5μm
d
131-*Malus zumi* 'Calocarpa'
area of pollen equatorial plane=1
$a_1>1/8$
$a_2>1/8$
$a_3>1/8$
$3/8<A=a_1+a_2+a_3<6/8$
n=3
PRM
5μm
e
78-*Malus* 'Kelsey'
area of pollen equatorial plane=1
reticulate
IR
5μm
e
105-*Malus* 'Regal'
area of pollen equatorial plane=1
psilate smooth
IR
5μm
e
107-*Malus* 'Roger's Selection'
area of pollen equatorial plane=1
$a<1/8$
$a<1/8$
IR
5μm
e
111-*Malus* 'Royalty'
area of pollen equatorial plane=1
reticulate
IR
5μm
e
114-*Malus* 'Show Time'
area of pollen equatorial plane=1
rugulate
IR
5μm
e
115-*Malus* 'Snowdrift'
area of pollen equatorial plane=1
$a_{1\text{-}5}<1/8$
IR
5μm

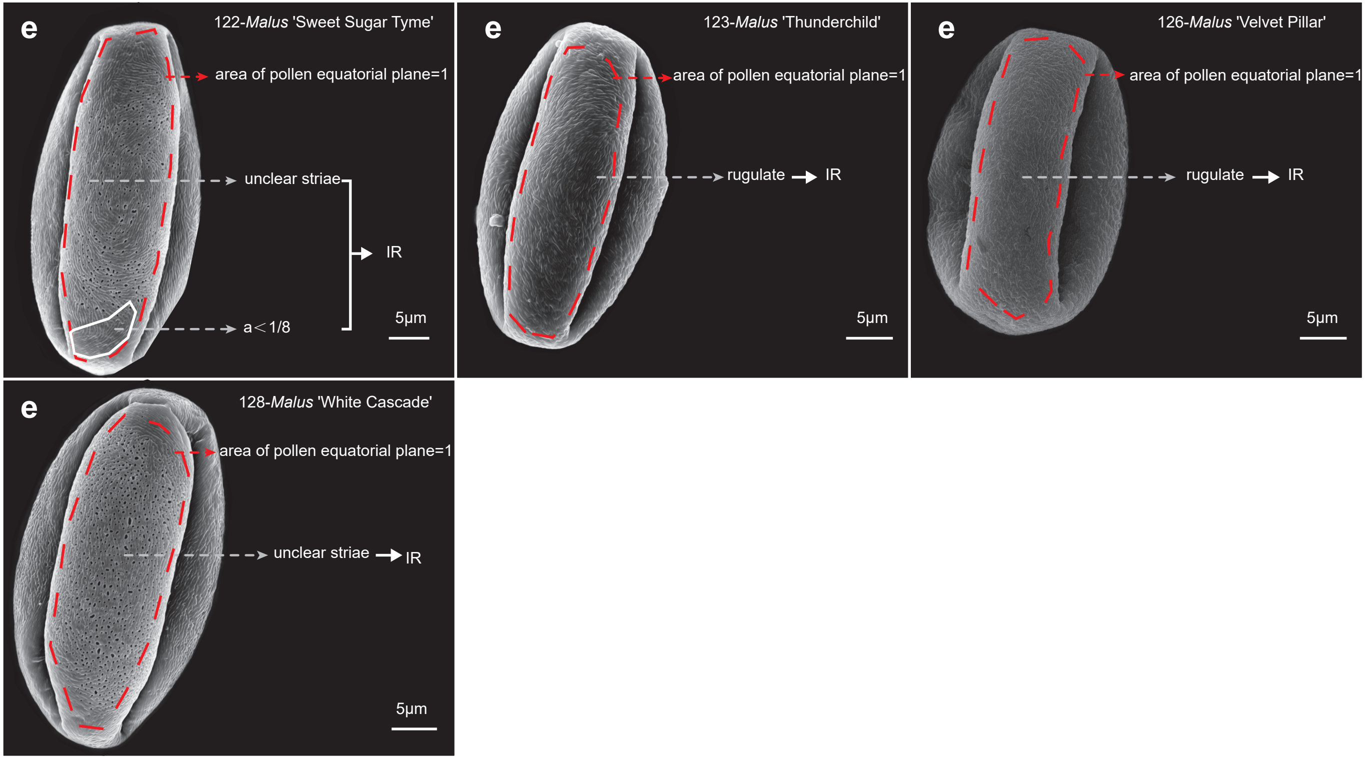
e
122-*Malus* 'Sweet Sugar Tyme'
area of pollen equatorial plane=1
unclear striae
IR
a<1/8
5μm
e
123-*Malus* 'Thunderchild'
area of pollen equatorial plane=1
rugulate
IR
5μm
e
126-*Malus* 'Velvet Pillar'
area of pollen equatorial plane=1
rugulate
IR
5μm
e
128-*Malus* 'White Cascade'
area of pollen equatorial plane=1
unclear striae
IR
5μm
